# One Health Landscape in Tennessee: Current Status, Challenges, and Priorities

**DOI:** 10.3390/tropicalmed10060150

**Published:** 2025-05-27

**Authors:** Walid Q. Alali, Jane Yackley, Katie Garman, Debra L. Miller, Ashley Morgan, Wesley Crabtree, Sonia Mongold, Dan Grove, Emily Leonard, Mary-Margaret A. Fill

**Affiliations:** 1 Department of Biostatistics & Epidemiology, College of Public Health, East Tennessee State University, Johnson City, TN 37614, USA; 2 Communicable and Environmental Diseases & Emergency Preparedness, Tennessee Department of Health, Nashville, TN 37243, USA; 3 One Health Initiative, University of Tennessee, Knoxville, TN 37996, USA; 4 Animal Health Division, Tennessee Department of Agriculture, Nashville, TN 37220, USA; 5 Wildlife & Forestry Division, Tennessee Wildlife Resources Agency, Nashville, TN 37211, USA; 6 School of Natural Resources, University of Tennessee Institute of Agriculture, Nashville, TN 37211, USA; 7 Office of Policy & Planning, Tennessee Department of Environment and Conservation, Nashville, TN 37243, USA

**Keywords:** one health framework, zoonotic diseases, climate change impacts

## Abstract

Tennessee’s ecological diversity, spanning forests, farmland, and urban areas, provides an ideal foundation for applying the One Health approach, which integrates human, animal, and environmental health. This review examines Tennessee’s current One Health landscape, highlighting active initiatives, ongoing challenges, and future directions. Key efforts involve workforce development, disease surveillance, outbreak response, environmental conservation, and public education, led by a coalition of state agencies, universities, and the Tennessee One Health Committee. These programs promote cross-sector collaboration to address issues such as zoonotic diseases, climate change, land use shifts, and environmental contaminants. Notably, climate-driven changes, including rising temperatures and altered species distributions, pose increasing threats to health and ecological stability. Tennessee has responded with targeted monitoring programs and climate partnerships. Education is also a priority, with the growing integration of One Health into K–12 and higher education to build a transdisciplinary workforce. However, the state faces barriers, including limited funding for the One Health workforce, undefined workforce roles, and informal inter-agency data sharing. Despite these obstacles, Tennessee’s successful responses to outbreaks like avian influenza and rabies demonstrate the power of coordinated action. To strengthen its One Health strategy, the state must expand funding, formalize roles, improve data systems, and enhance biodiversity and climate resilience efforts positioning itself as a national leader in interdisciplinary collaborative solutions.

## 1. Introduction

The One Health approach is a transdisciplinary approach that underscores the interconnectedness of the health of humans, animal, plants, and their shared environment. It fosters collaboration across diverse sectors, including human medicine, public health, veterinary medicine, environmental science, and beyond, to address complex health challenges holistically [1]. The One Health framework operates effectively at local, regional, national, and global levels [2] recognizing that risks at the human–animal–environment interface can vary significantly by state and region across the United States (U.S.).

Tennessee (TN) is characterized by its diverse geography, which includes mountains, forests, rivers, and agricultural lands. This variety supports a rich biodiversity of wildlife and domesticated animals, contributing to the state’s complex ecological network. The state’s economy is significantly supported by agriculture (about 4% of the USD 382.1 billion state gross regional product [GRP] in 2019), with livestock and poultry farming being prominent [3]. Additionally, TN has several urban centers where population density and industrial activities pose their own health and environmental challenges. The major One Health challenges in TN include emerging zoonotic diseases, vector-borne diseases, environmental contaminants, food safety and security, impact of changes in climate and land use, and biodiversity conservation. All these challenges have an impact on the livelihood of people in the state. Tennessee, like many states, has developed its own One Health initiatives to address such complex health issues at the local level. Addressing these health issues requires multisectoral transdisciplinary collaboration, coordination, and communication between state stakeholders to develop One Health action plans. Initiatives promoting research, disease surveillance, education, and policy development are essential for mitigating the impact of these interconnected health challenges. By fostering collaboration across various sectors such as public health, veterinary medicine, wildlife management, emergency preparedness, and environmental sciences, One Health initiatives aim to enhance disease surveillance, improve response capabilities, and promote sustainable practices.

The aim of this manuscript is to provide a review of the One Health landscape in TN, including the existing One Health initiatives, priorities, strengths, challenges, and gaps. Moreover, this manuscript will showcase the significance of the One Health approach through the collaborative work completed and/or ongoing in TN and will discuss the opportunities and challenges associated with One Health implementation in TN.

## 2. Status of One Health in Tennessee

### 2.1. One Health Initiatives

Tennessee has been actively involved in various One Health initiatives. The goals of the current One Health initiatives are centered around creating a healthier state, region, and nation by recognizing and addressing health issues at the human–animal–environment interface.

The One Health initiatives in TN are mostly housed in and directed by state-government agencies, the Tennessee Department of Health (TDH), the Tennessee Department of Agriculture (TDA), the Tennessee Wildlife Resources Agency (TWRA), the Tennessee Emergency Management Agency (TEMA), and the Tennessee Department of Environment and Conservation (TDEC), and academic institutions (University of Tennessee [UT], and East Tennessee State University [ETSU]). These initiatives can be classified into six broad categories: (1) workforce/capacity building development initiatives; (2) education, training, and research initiatives; (3) disease monitoring and surveillance; (4) environmental health and conservation; (5) rapid response to emerging health threats; and (6) public awareness and community engagement. In addition, initiatives that foster statewide interdisciplinary collaboration and cross-sector partnerships, such as the TN One Health Committee, exist. Details of the ongoing and future One Health initiatives are discussed in the following sections.

### 2.2. One Health Workforce

Professionals in human, animal, and environmental health sectors make up the One Health workforce in TN. Partnership and collaboration between the professionals at local, state, and federal government agencies, organizations and universities are facilitated in part by the TN One Health Committee which serves as a starting place for One Health responses, advocacy, and outreach. Agencies participating in the TN One Health Committee include the TDH, TDA, TWRA, TEMA, TDEC, UT, and ETSU and other partners.

A major challenge for One Health is the lack of dedicated workforce and funding. While the functional One Health workforce is made up of individuals from organizations involved in human, animal, plant, and environmental health, most agencies do not have a position or role dedicated to One Health. This lack of clarity can make it challenging to identify key stakeholders in organizations and ensure staff have bandwidth to support One Health projects outside their standard job duties. Establishing One Health positions or titles could help identify key players for One Health initiatives and show agency commitment to interdisciplinary health work.

The TN One Health Committee is managed without any dedicated funding for staff or meetings. This makes the committee vulnerable to staff changes, conflicting priorities and funding instability at each agency. To strengthen the committee, TDH established a planning subgroup, called the One Health Planning Committee, to build in additional capacity for leading and managing the One Health Committee. This group expands the number of staff and agencies involved in planning meetings and managing the One Health Committee, making it less vulnerable to staff turnover and changing priorities.

Funding for One Health is limited and is often project- or agency-specific. For example, TDA maintains limited One Health funding to support field projects and a portion of a veterinarian position. However, most agencies do not receive One Health funding. Agencies will need to think creatively about the use of funds for One Health projects and identify opportunities to advocate for dedicated funding to support initiatives into the future.

### 2.3. One Health Education/Training Programs

#### 2.3.1. K-12 Programs

Incorporating One Health principles into education is essential for fostering a comprehensive understanding of the interdependence between human, animal, plant, and environmental health [4]. By integrating One Health concepts into curricula at all levels, students can develop a holistic perspective of complex health challenges and be better equipped for devising potential solutions. However, applying a One Health approach requires knowledge-based skills; thus, it is essential to initiate transdisciplinary literacy and practice teamwork from childhood. Engaging K-12 students in One Health topics such as biodiversity, ecosystem services, zoonotic diseases, and climate change can activate critical thinking and problem solving. The UT One Health Initiative (UTOHI) has partnered with UT Extension, UT Gardens, TDH, TDA, Biology in a Box, Nashville Zoo, 4-H, and Lincoln Memorial University to develop publicly available, K-12 education materials [5] These materials include lesson plans and leader guides (including PowerPoint slides) addressing the TN Standards of Learning, covering One Health topics, and incorporating cases (like the global SARS-CoV-2 pandemic) toward which students can apply learned One Health concepts [6,7,8]. Further development and sharing of communal One Health curricula can ease the burden on educators interested in teaching a One Health approach to their students. Incorporating One Health into education promotes a sense of responsibility and stewardship towards our planet and its inhabitants. Students learn to recognize the impact of human activities on ecosystems and biodiversity, as well as the reciprocal influence of environmental health on human and animal well-being. By integrating environmental education into early learning curricula, children can grow up with an appreciation for the interconnectedness of food systems, energy use, and water resources. This foundational understanding primes them for more complex concepts, including the application of a One Health approach, as they advance in their education and eventual careers.

#### 2.3.2. Collegiate Level Training

Targeting undergraduate curriculum may be one area to engage students with diverse interests [9]. One study articulates that, “to acculturate planetary level thinking, One Health education should start early in the undergraduate years and draw upon multiple teaching traditions and knowledge systems to create an effective, reinforcing cycle of transdisciplinary thinking and holistic approaches” [10]. UTOHI advocates for the construction of One Health minor programs to help impart the importance of this transdisciplinary thinking. Therefore, we need experts structuring One Health curricula as a minor for undergraduate students to allow the preservation of forming experts in various disciplines while enhancing their abilities to work collaboratively with others. Students have a primary area of academic focus while taking classes toward their One Health minor which requires them to stretch beyond the confines of their major. The undergraduate One Health curricula at the UT includes competencies in the One Health approach such as communication and leadership, translation of evidence to policy, global issues, and cross-disciplinary science, and provides opportunities for independent capstone projects or undergraduate theses. Additionally, by structuring the One Health curricula as a minor (and not a major), diverse and inclusive student cohorts are created. Others have noted that, “It is in such mixed classes that disciplinary thinking, world views, life experiences, cultural backgrounds, and knowledge systems can be shared” [10].

#### 2.3.3. Post-Baccalaureate Training

Capturing those beyond undergraduate training is also crucial for devising comprehensive and collaborative solutions to complex problems [11]. In some cases, with nursing, veterinary medicine, human medicine, public health, and other health-related graduate programs, for example, it may be more appropriate to offer One Health curricula in the form of a certification program [10]. A graduate certificate in One Health and Climate Studies at ETSU provides students with a broad overview of how climate change is impacting the global environment, and how this will, in turn, impact human, animal, and environmental health and well-being. Offering these certificates via distance learning may increase accessibility to health professionals [12], but we encourage the incorporation of in-person service learning and practicum components to apply the learned One Health concepts. Assignments should incorporate critical thinking strategies that challenge students to consider diverse perspectives and develop holistic solutions to further enhance their ability to engage effectively with the local community needs. Graduate students equipped with this knowledge are better prepared to address real-world challenges through informed research that resonates with both local cultures and regulatory frameworks.

For early professionals and professionals advanced in their careers, learning, practicing, and applying the One Health approach may involve training in the form of continuing education courses [13]. In response to the Quadripartite’s call for ‘cross-sectoral competencies’ in their Joint Plan of Action on One Health [14], authors, on behalf of the Network for Ecohealth and One Health (NEOH), have proposed an updated set of One Health core competencies [15]. These competencies include skills- effective communication, collaborative and resilient working, systems understanding; values and attitudes—transdisciplinary, social, cultural, and gender equity and inclusiveness, collective learning, and reflective practice; and knowledge and awareness—One Health concepts, theoretical and methodological pluralism, harnessing uncertainty, paradox, and limited knowledge [15]. Using these core competencies as a framework, we can create quality One Health training materials to build and maintain a large and appropriately trained One Health workforce which is critical in assuring the health of people, animals, plants, and the environment [12].

Regardless of education or career stage, One Health training is crucial for answering the world’s most complex health problems. By providing accessible and broadly applicable education and continued training in the One Health approach, graduates are better equipped to address the ongoing (and ever-emerging) challenges that face the planet today. Furthermore, by emphasizing the interdependence of human, animal, plant, and environmental health, educational initiatives can inspire individuals to adopt sustainable practices and advocate for policies that promote global health.

### 2.4. Cross-Cutting Strengths

Tennessee’s combination of targeted teachings and research across all levels of education with a focused drive of governmental agencies for collaboration and open communication has created a unique opportunity to rapidly advance the One Health agenda of the state and its preparedness for current and future emerging disease incidents. The One Health Committee, an interagency assembly, has helped foster joint opportunities between Tennessee’s government agencies and academic institutions, including a partnership between the State Public Health Laboratory, the Kord Animal Health Diagnostic Laboratory, and the UT-Institute of Agriculture for laboratory testing support with potential zoonotic or rabies infections, as well as interagency information sharing about public health risk or vector-borne diseases, and disease tracking and emergency preparedness planning efforts. One important benefit of this union was promoting the creation of the Animal Emergencies Task Force, a critical working group tasked with compiling resources and developing guidance and response plans to minimize the environmental, animal, and resultant human health impacts of natural disasters and emerging diseases.

The main strength of these cooperative endeavors may best be demonstrated through the breadth of partners involved in the 2024 Movement Standstill Exercise conducted by TDA. During this exercise, members of TDA collaborated with the TDH, TEMA, TWRA, TDEC, state law enforcement agencies, and multiple other industry and government partners to evaluate the logistics involved and resources needed to conduct and enforce a 72 h animal movement standstill in the event of a critical foreign animal disease introduction or outbreak. Such an event could be devastating to animal agriculture, the environment, and the human food supply, but as the exercise demonstrated, these impacts can be minimized and the health of all promoted through diligent, open communication and collaboration.

## 3. Top Priority One Health Actions in TN

### 3.1. Cross-Sectorial Data Sharing

Cross-sectorial data sharing is a critical component of One Health initiatives. Effective data sharing among different sectors is essential for addressing complex health issues that span human, animal, and environmental health. In TN, state agencies and academic institutions are working together to establish robust data-sharing frameworks that support One Health goals. Through collaboration, coordination, and communication among various state agencies and academic partners, TN can leverage data-driven insights to improve health outcomes across the three domains. These efforts facilitate timely and informed decision-making, enhance research capabilities, and improve health outcomes for all. Table 1 shows the key agencies and their core roles. Table 2 shows the proposed benefits of cross-sectorial data sharing.

The proposed implementation strategies to enhance One Health data collaboration include establishing formal data-sharing agreements between state agencies and academic partners that define data types, security measures, and access protocols. Collaborative data systems and online platforms will be developed to enable standardized, integrated data exchange and analysis across agencies, with user-friendly interfaces and analytical tools. Regular stakeholder meetings, such as the May 2024 hybrid One Health meeting in Nashville, TN, will foster communication, trust, and coordinated planning. Additionally, training and capacity building—through workshops and academic programs—will equip current and future professionals with the necessary skills in data management, analysis, and platform use, ensuring sustained support for data-sharing initiatives across sectors.

### 3.2. Cross-Sectorial Collaboration and Communication

#### 3.2.1. State and Federal

Interagency and cross-sectorial collaboration between state and federal partners is vital to TN One Health, disease response, and emergency management strategies. The professional relationships and cooperative agreements developed through these interactions are utilized daily to share subject matter expertise and invaluable disease tracking data through communication between the TDH and Centers for Disease Control and Prevention (CDC) or TDA and the United States Department of Agriculture (USDA). These partnerships also aid in the performance of daily regulatory tasks through the sharing of field staff responsibilities and the preparation for and response to potential disease outbreaks and natural disasters. One such circumstance would be when facing an animal disease outbreak, such as avian influenza in poultry and most recently in dairy cattle [16,17]. During these occurrences, veterinarians and animal health technicians of the TDA collaborate daily with their federal partners in the USDA to communicate risks and management of infectious disease cases, contain the spread of pathogens, and minimize loss of animals and resources. These agents assist each other with case management, incident response, and plan development and implementation while also communicating any human disease risk to the TDH and CDC. While working together in the field for disease response and control, the USDA also aids the state public health and animal disease labs with facilitating surge disease-testing needs through the National Animal Health Laboratory Network and offering financial assistance for diagnostic or advanced genetic testing [18]. These coordinated actions are guided and managed by the Joint USDA-TDA Incident Management Team and are key to the successful control of animal disease outbreaks and maximizing the health of all the animals, humans, and environments involved. Moreover, USDA-Wildlife Services and TWRA coordinate to share information and respond to reports of mortality/morbidity events in wild birds in TN.

#### 3.2.2. Academic, NGOs, Private-Sector Partnerships, and Local Communities

Non-governmental organizations (NGOs) play a vital role in advancing the One Health approach by addressing complex, multifaceted challenges at the community level and bridging gaps between research, policy, and practice. They help connect evidence-based solutions with stakeholder needs and contribute to both problem identification and the development of practical, sustainable interventions. In TN, NGOs such as The Nature Conservancy, the Humane Society, Remote Area Medicine, and locally founded organizations like the Children’s Foundation Research Institute and Dolly Parton’s Imagination Library offer unique perspectives and resources. NGOs promote One Health through advocacy, raising awareness about the interconnectedness of human, animal, plant, and environmental health. They also engage in research, build local capacity through training and education, facilitate cross-sector partnerships, and support disaster response efforts. By fostering trust and empowering local communities and vulnerable populations, NGOs are indispensable in implementing effective and inclusive One Health strategies.

People in local communities, especially low-income families, small-scale farmers, Indigenous peoples, and migrant workers, live and work closest to the animals and environments that shape their health. Because they see problems first-hand, they are often the first to notice when something is off, like strange illnesses in backyard chickens or sudden fish die-offs. Their on-the-ground knowledge fills in details that outside experts can miss, helping refine risk estimates and guide quick action. When these groups have an equal say in planning solutions, whether it’s a vaccination drive or a safe-water project, participation rises, and the fixes actually fit local customs and budgets. In short, bringing vulnerable communities into One Health makes it a shared, equity-focused effort that protects people, animals, and the planet all at once.

### 3.3. Outbreak/Pandemic Preparedness and Response

One Health partnerships are necessary for pandemic preparedness and response. Partnerships were leveraged during the COVID-19 pandemic response. A key strength of the pandemic response was routine communication across the agencies related to current and planned response strategies. TDH, TDA, and TEMA teams joined routine calls to debrief recent activities and give situational awareness updates to partners. Response to highly pathogenic avian influenza (HPAI) also required partnerships between numerous agencies involved in the human, animal, and environmental health response to positive poultry flocks in TN. In 2017 and 2022, TN responded to multiple detections of HPAI in poultry, which required coordination between TEMA, TDA, TDH, and federal partners.

### 3.4. Animal Emergencies and Emerging Zoonotic Disease

TDA established a HPAI Task Force in 2015, in collaboration with TDH and TEMA, to prepare and respond to the previous national disease outbreak in domestic poultry. In 2023, TN re-established and updated it as an Animal Emergencies Task Force with the purpose of being an All-Hazards Animal Emergency preparedness and response collaborative group, including responses to emerging zoonotic diseases. A wide variety of TN-based agencies were included representing a diversity of expertise including NGOs and private sectors. Participants included TN agencies responsible for emergency management, agriculture, human health, wildlife, environment and conservation, safety and homeland security, human services, government general services, commerce and insurance, military, and the TN Poultry Association, UT, and the USDA. Task force subgroups include biosecurity, depopulation, disposal, communication, zoonotic/public health, and finance. The task force has created greater interagency awareness of capacity and responsibility, allowing for the quick implementation of new initiatives, such as the incorporation of TDH public health presence on site during avian influenza responses.

Animal emergency planning also includes Disaster Animal Response Teams (DART) and functional exercises. The DART program is a state-wide program facilitated by the State Veterinarian’s office at TDA. The program was established in the 1990s by TDA to provide a statewide standardization for credentialing animal emergency responders and has expanded as needs and emergencies have surfaced. TDA has also facilitated numerous planning exercises, including an Emergency Animal Standstill exercise in 2024, which brought agencies together to plan for a situation where a potential foreign animal disease is identified, and all animal movement must be halted for 72 h to investigate the illness and its impact.

### 3.5. Zoonotic Disease Prioritization

TDH is in the early stages of planning a One Health Zoonotic Disease Prioritization workshop that will bring together multidisciplinary partners from human, animal, and environmental health and other sectors. The goal of the workshop is to prioritize zoonotic diseases of greatest concern and to develop action plans for addressing priority diseases.

## 4. Role of Research and Outreach

Despite numerous calls to apply a One Health approach in research, wholly multidisciplinary teams are infrequently actualized [2]. However, big problems require big teams. It is crucial for those teams to have representation from diverse entities, like academic universities, research institutions, industry, and the public at large to aid in research design and the effective dissemination of findings [2]. Building partnerships is the first step in conducting meaningful interdisciplinary research. Bridging the gap between academic researchers and the breadth of stakeholders requires intentionality and effective communication from the onset of any research endeavor. Early collaboration is essential for a One Health approach to be effectively utilized in research, and it has been shown to be a strong predictor for the progression and success of early-career professionals [19]. Additionally, access to the scientific literature by the One Health community is still limited to open access journals, particularly access to the animal health literature [20]. One of the major challenges to creating efficient and diverse interdisciplinary teams is that traditional funding streams and limited budgets often limit collaborative capacity. This is the case for TN research efforts and efforts beyond. Additionally, training grants (funding for graduate students and post-doctoral associates) can be extremely beneficial for research teams to take on and accomplish large projects as well as serving to train the workforce, but these types of grant programs should be further bolstered within the state. UTOHI serves as a conduit and catalyst for these types of partnerships by linking researchers and stakeholders. Through partnerships, like those with the Oak Ridge National Laboratory (ORNL), the National Institute for Modeling Biological Systems (NIMBioS), TN RiverLine, the Smith International Center, TDH, TDA, and many others, UTOHI helps facilitate teams equipped to tackle local, national, and global problems. Additionally, UTOHI has created working groups (and accompanying interactive webpages) for antimicrobial resistance as well as chronic wasting disease, and environmental contaminants and toxicology to help facilitate diverse group discussion and research collaboration around these important One Health topics. Moreover, the current ETSU One Health research is focused on the epidemiology of antibiotic resistance at the human–animal–environment interface, including studies on antibiotic-resistant bacteria prevalence and distribution in the environment (in water and wastewater) as well as in clinical settings (humans and animals). This research is being conducted in collaboration with regional healthcare systems, TDH, and TDA. The creation and networking of similar One Health-focused entities is important in helping multidisciplinary teams function effectively and frequently in research.

## 5. Examples of One Health Approach in TN

### 5.1. Outbreak Investigations and Other Cases Studies

#### 5.1.1. HPAI Outbreak

Between September 2022 and January 2023, TN had nine separate confirmed cases of highly pathogenic avian influenza in domestic poultry spanning five counties in west, middle, and east TN. During this period, TDA and USDA staff worked side by side with industry, academic, and government partners to enact quarantines and control zones, inform the public of animal movement restrictions, investigate potentially affected flocks, sample animals for confirmation testing and movement permits, depopulate and dispose of infected animals, and return farms to safe, sanitary conditions where new animals could be raised. This time- and labor-intensive process required not only the collaborative efforts of TDA and USDA personnel, but numerous other governmental and academic agencies, including volunteer technicians from Kord Animal Health Diagnostic Lab who provided off-hour services as needed to run samples for emergency cases, TDH-contacted persons exposed to potentially infected birds, such as flock owners and on site responders, to help ensure there was no transmission from birds to humans, the TDEC helped provide guidance on disposal methods to manage the environmental impact on water tables and the land from animal burial, the USDA Wildlife Services aided in depopulation and wild bird surveillance, the TEMA provided communication equipment for rural areas along with local logistical support, and UT Extension agents acted as subject matter experts for disposal of animals to make sure all infectious material was removed appropriately. Only through the collaborative efforts of all these agencies, institutions, and industry partners, was TN able to contain the outbreak and dispose of almost 418,000 infected birds safely. This collective endeavor subsequently led to the re-formation of the TN HPAI Task Force and modification to become the Animal Emergencies Task Force to prepare for and help minimize the human, animal, and environmental health impact of all types of animal emergencies.

#### 5.1.2. Salmonella Control in Live Poultry

An example of interagency partnership between TDH, TDA, and CDC to respond to and control outbreaks of salmonellosis associated with live poultry (LP) is provided here. *Salmonella* lives in the intestinal tract of LP. The bacterium is also found in the feces of LP and can contaminate portions of their body such as their feathers, feet, beaks, and their living environment (i.e., bedding, coops, etc.). Though LP usually do not become sick with *Salmonella*, they can spread the bacteria to people which can cause disease. During 1990–2014, there were a total of 53 LP-associated *Salmonella* outbreaks in the U.S. These outbreaks were associated with 2630 salmonellosis cases, 387 hospitalizations, and 5 deaths. The number of LP outbreaks reported annually has increased substantially in recent years. During 2015–2022, there were 88 multistate LP-associated salmonellosis outbreaks and 7866 outbreak-associated cases in the US with 30% of cases hospitalized and 9 deaths [21]. TN residents accounted for 3.8% (296/7866) of these outbreak-associated cases including a higher proportion of hospitalizations (38%; 91/240) and one of the nine deaths across the US. Only a small proportion of *Salmonella* infections are diagnosed and reported, meaning the actual number of cases in these outbreaks is likely much larger [21]. To respond to these outbreaks, TDA and TDH work together to conduct environmental sampling and testing at retail locations where LP were purchased by the human cases in the current year’s outbreaks and at the cases’ residences. TDA and TDH provide safe LP handling education to retailers and cases. If the outbreak strain is isolated at the retailer or case’s residence, TDA and TDH work together with CDC to conduct traceback to identify the hatchery from which the LP originated. TDH and TDA also conduct traceback by contacting TN retailers where cases in outbreaks reported purchasing LP to identify potential source hatcheries for those purchases and share that information with CDC. Hatcheries are notified by CDC, along with public health and agricultural agencies from the state where the hatchery is located, when LP from their facilities are associated with *Salmonella* outbreaks and are provided with best practices for preventing *Salmonella* transmission. The relationship and the collaboration between TDA, TDH, and federal partners are critical in the response to LP outbreaks in TN and nationally. Without this ongoing collaboration, state and federal partners would not be able to identify impacted retail locations and hatcheries.

#### 5.1.3. Rabies Response

Rabies education, prevention, and response efforts in TN are managed by numerous agencies, including TDH, TDA, TWRA, animal control agencies, and USDA—Animal and Plant Health Inspection Service (USDA-APHIS) Wildlife Services. TDH maintains the rabies statutes, which require rabies vaccination for dogs, cats, and ferrets, clarifies the necessary response to animal bites, and outlines the role of veterinarians and animal control in rabies control efforts. The TDH Environmental Health program provides rabies vaccination tags to veterinarians across the state to track vaccination efforts [22]. TDH Environmental Health and local animal control agencies coordinate domestic animal observation for animals involved in a bite to determine the risk of rabies transmission. The TDH Zoonotic Epidemiology team and local public health professionals provide rabies risk consultations. The team advises on the need for animal observation, the rabies post-exposure prophylaxis vaccination, and provides consultation to healthcare providers if there is a suspect human case of rabies. The TDH State Public Health Laboratory is responsible for testing animals to aid in public health decision-making. The TDH laboratories in Nashville and Knoxville test approximately 1000 animals for rabies every year. The laboratory uses the gold standard direct fluorescent antibody test, which requires the staining and observation of three cross-sections of the brain. Large animal specimens requiring testing (e.g., horse, cow) are prepared by the TDA Kord Diagnostic Laboratory and sent to the SPHL for testing. TWRA maintains the captive wildlife statue [23] which prohibits individuals from owning wildlife. TWRA also conducts training and approval for wildlife rehabilitators in TN, ensuring they are trained on rabies control and other relevant topics. Annually, TN participates in the USDA-WS oral rabies vaccination (ORV) campaign, which distributes vaccine baits for raccoons in eastern TN. This program aims to prevent the westward spread of raccoon variant rabies across TN and the U.S.; ORV baiting has occurred since the 1990s and has been a highly effective tool for managing the spread of raccoon rabies. Due to all of these efforts, the incidence of animal rabies continues to be low in TN (~30 animals/year). In 2023, TDH coordinated the first ever Rabies Interagency Meeting to bring together key members from TN agencies, federal agencies and veterinary schools involved in rabies control work. This meeting allowed for information sharing and discussion about challenges and solutions for rabies response. Continued interagency partnership will ensure TN can respond to and prevent rabies in the future.

## 6. One Health Challenges and Opportunities in TN

### 6.1. Emerging Zoonotic Diseases

Emerging zoonotic diseases pose significant challenges in TN, a state with diverse ecosystems and a growing population that increases human–animal–environment interactions. Tennessee’s agricultural sector, wildlife habitats, and urban expansion create conditions conducive to the spread of zoonotic pathogens such as rabies, tularemia, and avian influenza. Climate change, deforestation, and habitat fragmentation further heighten the risk by altering the distribution of vector species like mosquitoes and ticks, which transmit diseases like West Nile virus, Lyme Disease, and Rocky Mountain spotted fever. Addressing these challenges requires a robust public health infrastructure, interdisciplinary collaboration, and surveillance systems to monitor and mitigate vector-borne and zoonotic threats across human, animal, and environmental health domains. Public awareness and proactive measures, such as vaccination and wildlife management, are also critical in preventing outbreaks and minimizing the health impact on communities.

#### Zoonotic Diseases at the Domestic Animal-Wildlife Interface

The importance of the exchange of diseases at the human–domestic animal–wildlife interface is well documented and highlights the need for joint surveillance efforts. To fully understand the complexity that exists within the systems that lead to the human–domestic animal–wildlife disease interface requires a cross-disciplinary approach involving professionals from public health, veterinary health, wildlife health, agricultural economics, human dimensions, and wildlife management, to name a few.

As modern agriculture advances, the emphasis on biosecurity and preventing interaction between livestock, poultry and wildlife has become a primary goal. It is critical that state and federal agricultural and wildlife agencies participate in collaborative surveillance efforts on both sides of the fence or barn door and communicate within the state and across state lines to ensure the health and safety of both domestic and wild animals. HPAI is a timely example of such cooperative efforts. Wild bird surveillance efforts may serve as an early warning system of potential locations for outbreaks in poultry. Understanding when and where wild birds are migrating can help inform poultry producers to enhance biosecurity efforts and be on alert. Additionally, disease and parasite surveillance in livestock may help prevent the movement of zoonotic diseases into and throughout the state. Preventing the movement of diseased or infested livestock can prevent the potential of introducing disease into local wildlife populations that may serve as a reservoir for disease that spills back over into livestock or people (e.g., bovine tuberculosis in deer in Michigan, Asian Long-horned ticks in cattle in East TN). Once a disease becomes established in wildlife populations, it is nearly impossible to eradicate the disease from wildlife. Efforts to do so are extremely costly for both state agriculture and wildlife agencies. From 2006–2011, Minnesota spent a combined total of over USD 300 million to fight bovine TB that had spilled over into wild white-tailed deer [24]. It cost the dairy and cattle industry over USD 1 billion in lost revenue with many small producers having to completely shut down.

Tennessee has one of the fastest growing populations of people in the US. In 2000, it was estimated that TN had 5.6 million residents. In 2024, the estimate is 7.1 million residents, roughly a 27% increase in population. Population growth has increased the development of lands and areas that were previously uninhabited. This ever-increasing incursion into wild habitats has increased the interactions of both people and domestic pets with wild animals. Additionally, people moving to TN have come from a wide variety of areas, with diverse backgrounds and cultures. With this diversity comes a broad range of opinions relating to interactions with wildlife. In addition to domestic animal and human movements into TN, there has been an increase in invasive species that bring their own set of unknowns for those involved in One Health. Since the early 2000’s, nine-banded armadillos have slowly marched across TN. Armadillos in some areas of the southern U.S. have been shown to carry *Mycobacterium leprae*, the bacteria that causes Hansen’s disease (leprosy). It is not clear whether the northward march of armadillos has brought this zoonotic disease along with it [25].

The combination of changes in modern agriculture and rapid population growth in TN has led to many challenges for all involved in One Health, specifically at the domestic animal–wildlife interface when preventing disease transmission. It is clear that a combined effort is needed for the proper surveillance, monitoring, prevention, and communications as it pertains to zoonotic disease transmission.

In the near future, TDH plans to bring together key One Health partners to initiate a One Health Zoonotic Disease Prioritization workshop. The workshop will allow TN to prioritize the zoonotic diseases of greatest concern and to develop action plans to address each disease. The workshop structure will be similar to that of the U.S. Prioritization Workshop held in 2017 [26] and those held in many other countries. The diseases prioritized in the U.S. workshop were zoonotic influenza, salmonellosis, West Nile virus, plague, emerging coronaviruses, brucellosis, and Lyme disease. One Health partners including NGOs and private sectors in TN have also met as part of the Animal Emergencies Task Force led by TEMA to make plans for emerging conditions with zoonotic potential and One Health impact. This work primarily stemmed from HPAI response but has since expanded to include additional diseases of concern.

### 6.2. Emerging Contaminants

In alignment with the One Health approach, TDEC plays a critical role in addressing emerging contaminants (ECs) that pose risks to human and environmental health. These contaminants, which include harmful algal blooms (HABs), per- and polyfluoroalkyl substances (PFAS), pharmaceuticals, microplastics, and pesticides like neonicotinoids, present significant challenges due to their persistence, bioaccumulation potential, and complex pathways of exposure [27,28,29].

Addressing ECs requires substantial resources, interagency coordination, and adaptive strategies to keep pace with emerging scientific evidence. ECs like HABs and PFAS require sophisticated and consistent monitoring systems. Expanding such activities or adding new ones requires additional funding, staffing, and the need for more advanced analytical capabilities at scale to detect contaminants at trace levels. By integrating environmental monitoring with health and ecological assessments, TDEC contributes to a holistic understanding of how these contaminants affect interconnected systems, reinforcing the importance of a One Health framework in TN. TDEC is utilizing this holistic approach to address HABs and PFAS in the environment.

#### 6.2.1. Harmful Algal Blooms

HABs present a major One Health challenge, as they can release toxins that pose risks to human health, aquatic life, and animals that come into contact with contaminated water. Interagency partnership is critical for HAB surveillance and response in TN. TDEC and TDH began exploring harmful algal toxin occurrence in raw and finished water from community water systems in 2016 and established a TN HAB Interagency Workgroup comprising federal, state, and local agencies, universities, and public interest groups. In 2019, TDH began reporting HAB events and human and animal exposures to the CDC One Health HAB System [30]. HAB complaints, photographs, and details of exposure were solicited from the Interagency Workgroup, primarily by the U.S. Army Corps of Engineers, TN Valley Authority, TDEC and TDA. An initial Harmful Algal Bloom Response Plan was created with assistance from members of the Interagency Workgroup and public messaging about HABs was circulated via TDEC, TDA, and TDH social media and agency websites. TDEC began serving as the lead agency for HAB complaint response in late 2023. TDEC partners with TDH, TDA, and academic institutions to monitor water quality, assess ecological impacts, assess human and animal health impacts, and inform the public about HAB risks and mitigation strategies.

#### 6.2.2. Per- and Polyfluoroalkyl Substances

PFAS are a group of thousands of man-made chemicals that have been manufactured for a variety of consumer and industrial uses since the 1940s [31]. PFAS proliferate in the environment through historic, widespread use and PFAS chemicals breaking down very slowly over time due to strong chemical bonds. The epidemiological evidence suggests associations between the increase in exposure to (specific) PFAS and certain health effects [32]. TDEC’s ongoing response to PFAS includes implementing its PFAS sampling strategy for source water and reporting and publicly communicating the EPA’s Unregulated Contaminant Monitoring Rule (UCMR 5) results [33] and TDEC’s PFAS source water sampling findings [29]. Additionally, TDEC continues to raise awareness of PFAS exposure pathways and their potential impacts on human health and the environment through data and information collection, health education, and support for PFAS mitigation.

Through the statewide TDEC PFAS sampling strategy, TDEC is testing public drinking water sources for 29 PFAS compounds (the same 29 PFAS tested in EPA’s UCMR 5). As of 30 October 2024, TDEC has tested over 1243 source water samples for PFAS; full results of the sampling effort are expected in Spring 2025. TDEC continues to update interactive dashboards on TDEC’s PFAS webpage with sample results. TDEC developed interactive dashboards in 2024 to better communicate the findings of both TDEC’s PFAS source water sampling and EPA’s UCMR 5 sampling of finished drinking water [34] with the public [35]. TDEC continues to support research efforts to better understand the science of PFAS and continues to provide regulatory updates and PFAS information for public awareness and health education. TDEC continues to coordinate a multi-agency working group, in conjunction with TDH, TDA, TWRA, and UT’s Extension of the Institute of Agriculture, to address PFAS-related challenges and opportunities including ways to mitigate and remediate PFAS contamination in soil, water, and air [29].

Furthermore, TDEC supports sustainable waste management and recycling programs that reduce environmental contamination, protect wildlife habitats, and promote positive environmental and public health outcomes. These efforts highlight TDEC’s commitment to a One Health framework focused on tackling complex, interconnected health challenges across TN.

### 6.3. Changes in Climate

Changes in climate is a global concern declared by the World Health Organization as the biggest threat to humanity, and its impacts are becoming exceedingly visible with extreme weather events [36]. The southeast region of the U.S. experiences a range of extreme weather events including droughts, extreme heat, cold spells, hurricanes, floods, tornadoes, and heavy rainfall. The 2014 Intergovernmental Panel on Climate Change (IPCC) report predicted that these extreme weather events will become more intense, more frequent, and last longer [37]. Specific to TN, average temperatures have risen since the 1970s, and there has been increased variability in annual precipitation since the 1950s [38]. Some of these changes have been directly attributed to human land use like urbanization, but with an ever-growing population and development sprawling from city centers in TN, climate impacts may be increasingly driven by human activities (e.g., urbanization, deforestation, habitat alteration). For instance, in September 2024, Hurricane Helene had a significant impact on Northeast TN with widespread challenges affecting communities. The region faced substantial flooding, power outages, infrastructure damage, and disrupted transportation. Many residents were displaced due to the flooding of homes and businesses. Additionally, environmental concerns such as soil erosion and contamination of water sources have added to the challenges.

Climate projections for the southeast region show continued increases in mean annual temperatures (3–5 °F) for interior states like TN by 2050 with greatest warming during summer months along with an increase in the average annual number of days with maximum temperatures exceeding 95 °F. Annual precipitation is expected to decrease in some parts of the region but increase by up to 6% in TN by the end of the 21st century, with annual extreme precipitation days projected to increase in parts of TN by 2050 [38].

The outcomes of climate change have both direct and indirect impacts to the health of humans, animals, and the environment and undoubtedly should be addressed through the One Health framework. Extreme heat, for example, has been identified as the leading cause of weather-related deaths to humans in the U.S, and the annual number of heat-related deaths has continued to increase. Extreme heat (and other consequences of climate change like droughts, wildfires, and storm-related flooding) may also have cascading effects on human and animal health by impacting things like food security, or environmental health by leading to things like increased contaminated waterways from HABs [39].

Through a collaborative effort between TWRA, The Nature Conservancy (TNC), the National Wildlife Federation (NWF), a climate change vulnerability assessment was prepared as a supplement to the 2015 TN State Wildlife Action Plan and focuses on species vulnerability, potential vegetation change, and landscape feature resilience of TN [40]. The investigation found that 70 of the 119 (37%) species assessed were found to be “moderately vulnerable” or above with those species being from fish, mussel, and plant taxa. The factors that lead to the elevated classification included natural and anthropogenic barriers to dispersal, restricted habitat range, and high levels of physiological sensitivity to changing temperatures and moisture [38]. With respect to aquatic species, increasing temperatures and precipitation across the state will alter the hydrological conditions to which the species residing are adapted. These changes could be detrimental to survival of wildlife by limiting the ability to find adequate sources of food and shelter and limiting the ability to move between suitable habitats, inevitably impacting the rich biodiversity in TN.

Terrestrial environments in TN and species that rely on them will also continue to be negatively affected by climate change. Higher annual average temperatures are resulting in shifting species distributions and affecting host–parasite interactions. Many insects have increased survival over winter and are expanding distributions northward where they may find naïve populations of hosts. The southern pine beetle, for example, has had detrimental effects in TN, killing approximately 350,000 acres of pine forests by 1998 and leading to significant economic loss. The beetle is expected to continue to expand distributions northward, increase infestation rates, and increase the current annual pine mortality rate by 4–7.5-fold [41]. Similarly, the hemlock woolly adelgid is projected to outcompete and this will lead to a complete loss of eastern hemlock from the Cumberland Plateau and Mountains region by the end of the century [38].

The One Health networks in TN may begin to fill these knowledge gaps and support response planning. Through the TN One Health Committee, partner agencies have been able to coordinate monitoring and response to zoonotic diseases affecting avian species (i.e., West Nile virus, HPAI) by utilizing various monitoring tools (e.g., wildlife and agriculture field sampling, citizen observational reporting, human health monitoring), communicating results between agencies, and coordinating responses. The TN One Health Committee and Animal Emergencies Taskforce will need to continue to build upon these efforts to further understand risks to human and animal health and improve management options.

### 6.4. Non-Zoonotic Diseases/Loss of Biodiversity

Loss of biodiversity is a major concern of wildlife health managers. The full long-term effects of losing species to either disease outbreaks, the degradation and fragmentation of local ecosystems, or to toxins released into the environment is not known. Zoonotic diseases and diseases of agricultural importance receive the bulk of One Health surveillance efforts. Diseases impacting only one species or exclusively wildlife at times go unrecognized because the importance of the affected species and the link to the immediate impacts on human and domestic animal health may not be obvious. This disparity in emphasis is an artifact of how funding opportunities are prioritized and are made available for health surveillance efforts across the One Health community.

Those within the TN One Health community can recognize how the loss of millions of bats to the fungal disease, white nose syndrome (*Pseudogymnoascus destructans*), has the potential to negatively impact human and domestic animal health through the loss of an important insectivore species that removes millions of potential disease-carrying mosquitoes across our state each year. Additionally, the loss of a wide range of snakes due to snake fungal disease (*Ophidiomyces ophiodiicola*) may seem welcome to those who view snakes as vile vermin. However, snakes play an important role in pest management in local areas, removing rodents and other small unwanted “pests”. This loss of species diversity and its long-term impact on local insect and rodent population control may not be readily obvious to those making funding decisions. It is important for those involved in One Health to learn to communicate science with the decision makers so that issues like the loss of biodiversity and loss of species are more tangible concepts worth spending money to prevent all the negative ‘trickle down’ effects such as predator–prey imbalance, pollinator decline, disease outbreaks, habitat degradation, and further climate dysregulation.

A more tangible example of how a disease that impacts primarily wildlife species can impact people is chronic wasting disease (CWD), the prion disease of deer. A study conducted by the UT estimated the value of having Wildlife Management Areas across the state was worth over USD 576 million to the local economies [42]. The primary use of these areas is to provide public lands open to hunting. The species most hunted in TN is white-tailed deer. If a disease like CWD is introduced into an area, it may dissuade hunters from hunting in a given area. When that happens, people who hunt no longer travel to these areas, resulting in a loss of money flowing into the local economies (e.g., money being spent at hotels, gas stations, restaurants, local sporting goods stores, etc.). This feedback loop can lead to huge financial impacts on local communities and a decreased ability to manage the disease in deer through hunting in these areas. The ripples of fighting this one disease can be felt throughout all aspects of state wildlife agencies. Staff are often re-tasked to work on CWD surveillance and management efforts, and reducing the time and money spent on biodiversity and non-game species. Agencies also feel the impacts of people deciding to hunt less because they no longer purchase licenses or hunting equipment which form the backbone of conservation funding in the U.S.

In short, the overall impacts of non-zoonotic wildlife diseases and biodiversity loss can and do have far reaching impacts on One Health. The paths to understanding these impacts require a multi-disciplinary collaborative effort across health specialists, social scientists, economic specialists, communication specialists, wildlife biologists, and animal scientists. The growth of the TN One Health network is laying the foundations for many future avenues of understanding the importance of the linkages between multispecies and environmental health at the human–wildlife–domestic animal disease interface.

## 7. Recommendations

The One Health framework in TN is a multidisciplinary approach aimed at addressing the health interconnections between humans, animals, plants, and the environment. Current One Health initiatives in TN are spearheaded mainly by state government agencies and academic institutions, with an emphasis on workforce development, disease surveillance, environmental conservation, rapid responses to emerging threats, and public education. The One Health Committee serves as a central coordinating body, facilitating partnerships across public health, veterinary, and environmental sectors. Table 3 presents a set of recommendations that outline the next steps for strengthening One Health in the state.

## 8. Conclusions

Tennessee’s unique geographical and ecological diversity supports a rich network of wildlife and agriculture, posing both opportunities and challenges in zoonotic disease control, climate change mitigation, and biodiversity conservation. The state has taken proactive steps to address these challenges, including the creation of task forces and holding interdisciplinary workshops to prioritize emerging zoonotic diseases. However, challenges remain, such as the lack of dedicated funding, clear workforce roles, and comprehensive cross-sectorial collaboration. Limited budgets and resource constraints hinder full-fledged interdisciplinary teamwork and data sharing, which are critical for advancing One Health objectives.

Increasing investment in cross-sectorial collaboration, research, education, and capacity-building efforts will ensure that TN can continue to address its most pressing health challenges through the One Health framework.

## Figures and Tables

**Table 1:** Key One Health agencies and their core roles in Tennessee.

Agency	Core Role
Tennessee Department of Health (TDH)	Responsible for public health disease surveillance, disease prevention, and health promotion. The department collects and analyzes data on human health indicators and collaborates with other agencies to address zoonotic diseases, environmental health issues, and emergency preparedness and response
Tennessee Department of Agriculture (TDA)	Oversees agricultural practices, domestic animal health, and food safety. The department collects, analyzes, and reports data on animal health, pesticide usage, and crop conditions, which are crucial for understanding and mitigating risks to human and animal health.
Tennessee Wildlife Resources Agency (TWRA)	Manages wildlife populations and monitors wildlife health. The agency collects data on wildlife diseases, population dynamics, and habitat conditions and shares data with other agencies to inform public health strategies.
Tennessee Emergency Management Agency (TEMA)	Responsible for preparing for, responding to, and recovering from emergencies that impact the health and safety of Tennesseans. By collaborating with agencies focused on human health, animal health, and environmental health, TEMA contributes to a comprehensive and integrated approach to emergency management.
Tennessee Department of Environment and Conservation (TDEC)	Responsible for enhancing the quality of Tennessee’s air, land, and water and to be stewards of the natural environment. The department provides valuable environmental health data and insights to other agencies essential for understanding and addressing health issues that affect humans, animals, and ecosystems.
University of Tennessee (UT)	Home to the One Health Initiative, College of Veterinary Medicine, and the UT Institute of Agriculture, which contribute valuable data on animal health, agricultural practices, and environmental impacts. UT collaborates with state agencies to integrate veterinary and environmental data into public health initiatives
East Tennessee State University (ETSU)	Conducts interdisciplinary research on One Health topics, including zoonotic diseases, environmental health, and public health. ETSU collaborates with state agencies to share research findings and data, enhancing the overall understanding of health challenges in the region

**Table 2:** Proposed benefits of inter-agency cross-sectorial data sharing in Tennessee.

One Health Area	Benefit of Cross-Sectorial Data Sharing
Enhanced Disease Surveillance	Integrating data from human health, animal health, and environmental monitoring systems improves the ability to detect and respond to emerging zoonotic diseases. This comprehensive surveillance approach allows for early warning and rapid intervention, reducing the spread of infectious diseases.
Improved Public Health Responses	Data sharing enables a coordinated response to public health threats. For example, during an outbreak of a zoonotic disease such as HPAI, data from veterinary sources, wildlife monitoring, environmental assessments, and human health can provide a more complete picture of the situation, informing targeted interventions.
Informed Policy and Decision-Making	Access to shared data sets allows policymakers to make evidence-based decisions. Cross-sectorial data sharing provides a holistic view of health issues, ensuring that policies address the interconnectedness of human, animal, and environmental health.
Advanced Research and Innovation	Academic institutions benefit from access to state agency data, which can enhance research projects and lead to new insights and innovations. Collaborative research initiatives can address specific health challenges in TN, contributing to the One Health knowledge base. For instance, ongoing research on antimicrobial resistance in water sources between ETSU and TDH to address the environmental contribution of drug resistant bacteria to human health.
Resource Optimization	Sharing data across sectors prevents duplication of efforts and optimizes resource use. Agencies can coordinate their activities, share expertise, and leverage existing data to address health challenges more efficiently.

**Table 3:** A list of recommendations outlining a way forward to enhance One Health in Tennessee.

Subject	Area of Limitation	Proposed Solution
Dedicated Funding and Workforce	Lack of dedicated financial and human resources for One Health initiatives in TN	Advocate for sustained funding from both state and federal levels to support One Health training, data sharing, and interdisciplinary research efforts.
Cross-Sector Collaboration and Data Sharing	Lack of formal data-sharing agreements and systems between state agencies and academic institutions	To improve health outcomes, TN should continue to formalize data-sharing agreements and develop integrated data systems between state agencies and academic institutions. This would optimize disease surveillance and better inform decision-making.
Educational Programs	One Health education is limited to a few programs across the state	One Health education needs to be embedded more deeply at both K-12 and collegiate levels. Programs offered by UT and ETSU like the One Health minor and One Health and Climate Studies graduate certificate should be expanded to target health professionals in medicine, veterinary, and environmental sciences. Furthermore, academic institutions should collaborate with relevant state-agencies when developing One Health educational programs.
Targeted Zoonotic Disease Action Plans	A priority list of zoonotic diseases in Tennessee does not exist. In addition, there is a need for One Health action plans for rapid response to control zoonotic disease outbreaks.	Initiating workshops like the One Health Zoonotic Disease Prioritization would help TN to address priority diseases more effectively, including rabies, avian influenza, and salmonellosis, ensuring rapid response capabilities for future outbreaks.
Climate Change and Biodiversity Initiatives	Limitations related to cross-disciplinary work to address climate change impact on public, animal, and environmental health.	Addressing the intersection of climate change and One Health requires comprehensive studies into species vulnerability and shifting disease patterns. Cross-disciplinary teams should investigate how these factors impact human, animal, and environmental health in TN.

## Data Availability

No new data were created.
